# Inverse Method to Determine Fatigue Properties of Materials by Combining Cyclic Indentation and Numerical Simulation

**DOI:** 10.3390/ma13143126

**Published:** 2020-07-13

**Authors:** Hafiz Muhammad Sajjad, Hamad ul Hassan, Matthias Kuntz, Benjamin J. Schäfer, Petra Sonnweber-Ribic, Alexander Hartmaier

**Affiliations:** 1 Interdisciplinary Centre for Advanced Material Simulation (ICAMS), Ruhr-Universität Bochum, Universitätsstr 150, 44801 Bochum, Germany; hamad.ulhassan@rub.de (H.u.H.); BenjaminJosef.Schaefer@de.bosch.com (B.J.S.); alexander.hartmaier@icams.rub.de (A.H.); 2 Robert Bosch GmbH—Corporate Sector Research and Advance Engineering, 71272 Renningen, Germany; Matthias.Kuntz2@de.bosch.com (M.K.); petra.sonnweber-ribic@de.bosch.com (P.S.-R.)

**Keywords:** cyclic indentation, Vickers hardness, inverse analysis, numerical simulations, cyclic material properties, fatigue life

## Abstract

The application of instrumented indentation to assess material properties like Young’s modulus and microhardness has become a standard method. In recent developments, indentation experiments and simulations have been combined to inverse methods, from which further material parameters such as yield strength, work hardening rate, and tensile strength can be determined. In this work, an inverse method is introduced by which material parameters for cyclic plasticity, i.e., kinematic hardening parameters, can be determined. To accomplish this, cyclic Vickers indentation experiments are combined with finite element simulations of the indentation with unknown material properties, which are then determined by inverse analysis. To validate the proposed method, these parameters are subsequently applied to predict the uniaxial stress–strain response of a material with success. The method has been validated successfully for a quenched and tempered martensitic steel and for technically pure copper, where an excellent agreement between measured and predicted cyclic stress–strain curves has been achieved. Hence, the proposed inverse method based on cyclic nanoindentation, as a quasi-nondestructive method, could complement or even substitute the resource-intensive conventional fatigue testing in the future for some applications.

## 1. Introduction

Depth-sensing indentations or instrumented indentations are very useful means to characterize and determine mechanical properties (i.e., Young’s modulus and hardness) of thin films as well as of bulk materials [1,2,3,4,5]. Hyung [6] and Suresch et al. [7,8] have proposed two novel methods to identify the elastic modulus, yield strength, and the hardening exponent through nano-indentation. In addition, instrumented indentation experiments make it possible to determine further material properties, such as the strain hardening coefficient and yield strength [9]. Schmaling and Hartmaier [10] have introduced a method to identify plastic material properties (i.e., yield strength and work hardening rate) by using an inverse analysis for the remaining imprint after indentation. A comprehensive comparison of the hardness measurement approaches at diverse scales (i.e., nano, micro, and macro) of Brinell, Vickers, Meyer, Rockwell, Shore, IHRD, Knoop, and Buchholz was performed by Broitman [11]. He has not only described each indentation method but has also presented its inadequacies in evaluating results. Furthermore, he has discussed the effects of elasticity, plasticity, pileup, sink-in, grain size, and indentation size on determining hardness by means of depth-sensing indentation techniques at the micro- and nanoscale.

The applications of instrumented nanoindentation are not limited to the identification of conventional material properties, such as elastic modulus and hardness; the ease of performing this method has also attracted the attention of some authors who have employed it to estimate complex material properties like fatigue life or even the famous S-N (Wöhler) curve [12,13,14]. The determination of these quantities requires tedious and difficult conventional fatigue experiments; consequently, there are some analytical relationships available that allow the determination of the S-N curve and fatigue strength by using indentation hardness. For example, the Strzelecki model [12] has described the relationship between fatigue life and material hardness. Similarly, by utilizing the Murakami [13] formulation, Bandara [14] has presented full range S-N curves for six different medium steel grades. However, this suggested formulation is material-specific and requires two input parameters, i.e., Vickers hardness and ultimate tensile strength. Furthermore, Bandara [15] has used the Brinell hardness value in order to determine material fatigue strength. The three [12,14,15] aforementioned analytical approaches (mainly dependent on the hardness value of the material) have been comprehensively summarized by Strzelecki and Tomaszewski in [16], where they have expounded the merits and demerits of each individual approach and concluded that both models are good for predicting tensile strength and yield strength, while the Strzelecki model [12] is superior in the prediction of fatigue life as compared to the Bandara model [14,15].

In classical indentation experiments, only single loading and unloading on the specimen is performed for determining the desired mechanical properties. However, to study the fatigue life in materials, the cyclic indentation has attracted the attention of many authors [17,18,19,20]. For example, Lyamkin et al. [17] have shown the potential of cyclic indentation for studying the fatigue properties. As the cycles of indentations are repeated at the same location, the elastoplastic area of high cyclic indentation demonstrates fatigue under these conditions. For instance, a nanoimpact fatigue test has been studied by Faisal et al. [18] by using cyclic indentation, and they have concluded that the indenter geometrical shape (Berkovich or conical) and the indentation loading history are crucial in inducing film failure. Moreover, Haghshenas et al. [19] have employed cyclic nanoindentation in order to determine the indentation size effects and the strain rate sensitivity for tantalum. Similarly, Prakash [20] has demonstrated fatigue damage in materials with the help of two different nondestructive techniques, of which one is spherical cyclic indentation. In addition, he has investigated fatigue properties with a cyclic small punch test and with cyclic automated ball indentation and has concluded that the stiffness in the weld region drops more quickly in comparison to the base metal [21]. Xu et al. [22] have performed the numerical analyses of flat cylindrical indentation for polycrystalline copper. They have concluded that strain accumulation reached a steady-state indentation depth rate in sinusoidal cyclic loading just like in real fatigue experiments.

From this literature review, it is concluded that using instrumented indentation for determining the fatigue life of materials would reveal significant advantages of indentation testing (quasi-nondestructive technique, less time- and cost-intensive) over conventional fatigue experiments. Some analytical relations are indeed available to predict the S-N curve for fatigue life based on the hardness value, but these methods are mostly limited to steels and not applicable to softer materials, for instance, copper. Furthermore, it is not possible to analyze the microstructural influences. Hence, there is a need for a more general approach that can be applied to a larger range of materials. In addition, a method that uses the indentation experimental data for predicting complete uniaxial stress–strain hysteresis is still missing in the literature according to the authors’ knowledge. Yet, this uniaxial stress–strain hysteresis has a key role in fatigue life determination. Although some attempts have already been made, as discussed earlier, the literature still lacks a hybrid method that predicts the material fatigue life and the complete uniaxial stress–strain hysteresis with a combination of the numerical analysis and Vickers indentation.

## 2. Material and Experiment

### 2.1. Materials Specifications

In this study, we used a conventional quenched and tempered martensitic high-strength steel SAE 4150 (German denomination 50CrMo4, Judenburg GmbH, Judenburg, Austria) that exhibits a hardness of 38 HRC. The cyclic properties of this material were analyzed in detail by Schäfer et al. [23]. The second material used in this study, technically pure copper, is a conventional Cu-ETP R290 (Electrolytic Tough-Pitch, Wieland-Werke AG, Ulm, Germany).

### 2.2. Indentation and Fatigue Testing

**Figure 1:**
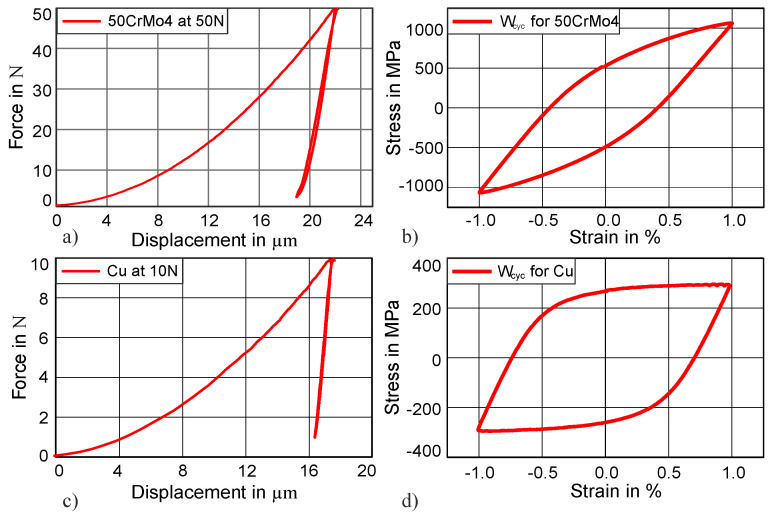
Experimental results for Cu and 50CrMo4: (**a**) indentation cycle at 50 N for 50CrMo4; (**b**) stress–strain from fatigue experiments of 50CrMo4; (**c**) indentation cycle at 10 N for Cu (**d**) Stress–strain from fatigue experiments of Cu.

Indentation testing was performed by using a small Zwick load indenter and was adapted from Kramer et al. [24]. The test was performed in a cyclic fashion; therefore, the indenter tip was repeatedly indented at the same spot. To hold the sample in the testing position, a constant minimal load remained between the cycles. A minimum load of 2 N and a maximum load of 50 N were used for martensitic steel. Further experiments were also performed for higher force amplitudes, i.e., at 75 and 100 N, and for higher hardness, such as 47 HRC at 50 N. In addition, a cyclic indentation test was performed for Cu with a maximum load of 10 N and a minimum load of 1 N. The load increased at a rate of 5 N/s. As an example of indentation testing, only the first complete cycle for 50CrMo4 and Cu is demonstrated in Figure 1a,c. Fatigue experimental data of uniaxial cyclic stress–strain curves were obtained from the study of Schäfer et al. [23]. Similarly, stress–strain hystereses are shown for the aforementioned two materials (50CrMo4 and Cu) in Figure 1b,d.

**Figure 2:**
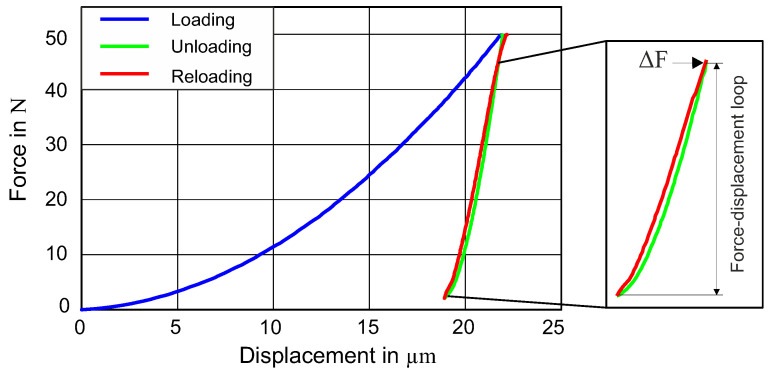
Force–displacement curve from the cyclic indentation curve. Components of the cycle are shown in different colors for clarity.

The complete cycle of the indentation test for martensitic steel (50CrMo4) is demonstrated in Figure 2 with three different colors. The first (blue) part of the curve indicates the loading, the green section displays the unloading, and the red portion demonstrates the reloading for the force–displacement. It can be seen that the unloading and reloading curves make a closed loop, which is named as the force–displacement loop (FD loop), and the intersection point is named ΔF. This loop will be used as a target curve for the parameter identification in the following section.

### 2.3. Numerical Models

**Figure 3:**
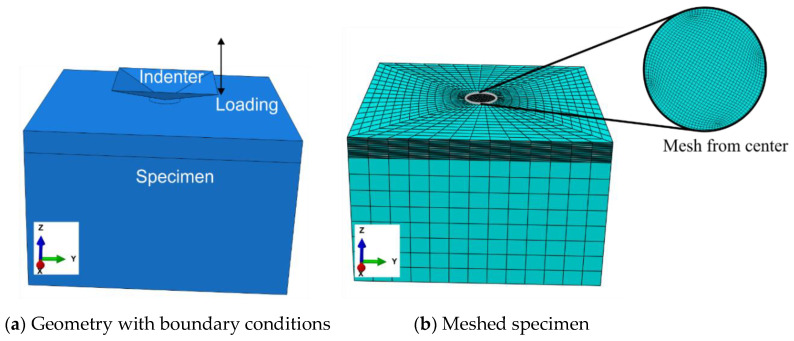
Details of the numerical model used. (**a**) Specimen is held fixed from the bottom and the indenter is placed in the center of the specimen that moves in and out during loading and unloading, respectively. (**b**) The fine meshing is performed at the center of the specimen.

The finite element model used in the investigation is depicted in Figure 3a. The indenter was modeled as a rigid body. The specimen was fixed from the bottom, and a vertical load was applied at the center by using a Vickers indenter. The square pyramid had an opposite face angle of 136° (DIN EN ISO 6507-2: 2005) [25]. For example, the applied force amplitude of 50 N in the simulation is explained as follows: The specimen was indented with the preselected applied force amplitude (i.e., 50 N), which was unloaded until the force of approximately 2 N and reloaded to the maximum force. Similarly, the simulations were run at other indentation force amplitudes of 25, 75, and 100 N. The force–time history from the experiments was used as an input for the simulations so that the loading in experiments could be fully depicted in the simulations.

It is our goal to identify the material parameters, which requires many simulations to be performed and is computationally very costly. Therefore, the size of the simulation model (2 mm × 2 mm and extruded to 1.5 mm) was optimized with a mesh size of 4 µm (Figure 3b); after the mesh convergence study, C3D8 linear elements with a full integration scheme were chosen for this paper. The friction effect between the indenter and the specimen was also studied, and it did not show a considerable effect on the simulation results in the scope of this work.

### 2.4. Material Model

In this investigation, the Chaboche material model was used, because it is one of the most efficient and convenient constitutive models incorporating the cyclic plasticity behavior of materials during cyclic loading. Furthermore, the ratcheting behavior of the material, which rises under cyclic loading, can also be analyzed with it. Details of this model for J2 plasticity are given in the subsequent section.

According to the von Mises yielding criterion [26], the yielding of material starts once the second deviatoric stress invariant J2 reaches a critical value. The common formula of this yielding criterion f is as follows:

(1) $$f=\sqrt{\frac{3}{2}\left(S-\alpha \right):\left(S-\alpha \right)}-\left(\sigma_{0}+R\right)$$

where $\sigma_{0}$, α, and *S* represent the initial yield stress, backstress, and deviatoric stress, respectively, and R represents the isotropic hardening (i.e., constant growth of yield surface) [27]. The increase in plastic strain with respect to the gradient of the yield surface will lead to the definition of the associative flow rule used in this study.

(2) $$R=Q\left(1-e^{-b\varepsilon_{eq}}\right)$$

where$\;\varepsilon_{eq}$ is the equivalent plastic strain, b determines the rate of isotropic hardening, and Q is the maximum change in the size of the yield surface [28,29].

In order to model the cyclic behavior of materials, a nonlinear kinematic hardening model was proposed by Armstrong and Frederick [30]. This kinematic hardening model contains only one backstress term (α) and was extended by Chaboche by decomposing the single backstress term into several backstress terms, making the Chaboche material model [28] capable of capturing the complex kinematic hardening behavior.

**Figure 4:**
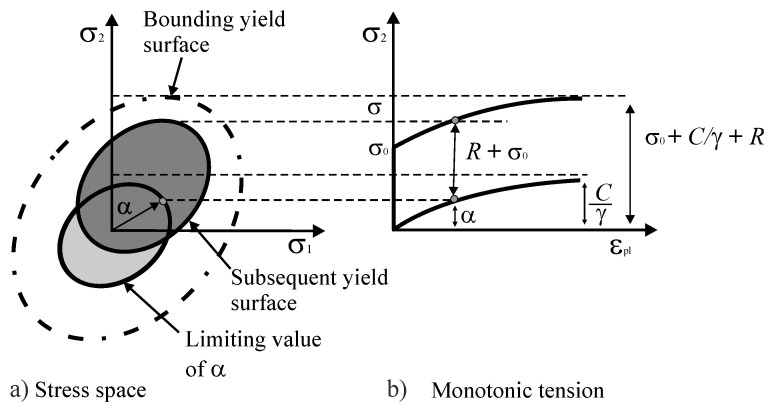
Graphical depiction of the combined hardening growth in the (**a**) stress space shown by the yield surface and (**b**) under monotonic tension presented as a stress–strain diagram, redrawn from [27] under the CC-BY license.

The decomposed backstress terms of Chaboche kinematic hardening [28] model are described in the following equation:

(3) $$\alpha =\overset{n}{\underset{i}{\sum }}\alpha_{i};\;d\alpha_{i}=\frac{2}{3}C_{i}d\varepsilon_{p}-\gamma_{i}\alpha_{i}d\varepsilon_{eq},$$

where $\gamma_{i}$ describes the reduction rate of the related modulus with respect to the plastic strain$\;d\varepsilon_{p}$, while *C_i_* represents the kinematic hardening moduli. The change in the yield surface of the combined hardening evolution for monotonic tension and in the stress space is graphically presented in Figure 4 [27].

In the present study, three backstress terms, which comprise six unknowns, and isotropic softening with two unknown parameters are used initially, leading to a total of eight unknowns which are identified by using an inverse modeling technique. Furthermore, an effort has been made to use only two backstress terms, which reduces the unknown terms to six with the almost same quality of results in our case [31]. Therefore, in this study, only the results of two backstress terms are shown.

## 3. Inverse Parameter Identification

**Figure 5:**
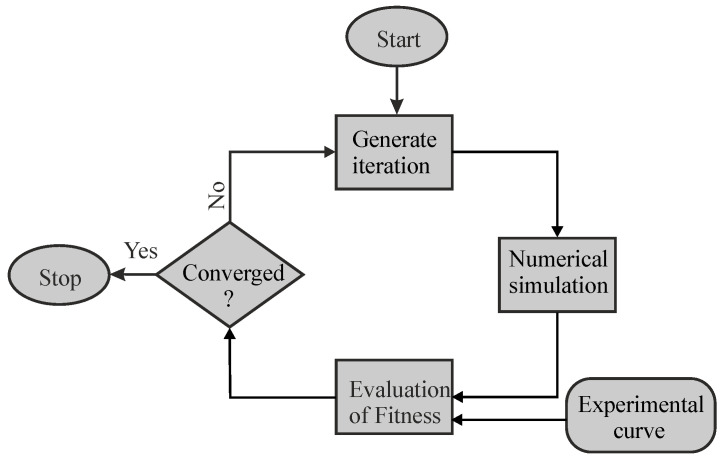
Optimization loop used to identify the material parameters by fitting the experimental curve with the simulation curve.

In order to capture the experimental force–displacement loop by simulation, the commercially available LS-Opt optimizer (DYNAmore GmbH, Stuttgart, Germany) [32] was used. The inverse identification technique was applied, where the difference between experimental and simulated values of the force–displacement loop, shown in Figure 2, was minimized by varying the material parameters in an iterative procedure, as shown schematically in Figure 5. The quality of fit between the simulated and the experimental force–displacement loops was evaluated by using the normalized mean square error (NMSE):

(4) $$\mathrm{NMSE}=\frac{1}{N}\underset{i}{\sum }\frac{{\left(E_{i}-S_{i}\right)}^{2}}{\bar{E}\bar{S}}\;,$$

an objective function, where $\bar{E}$ and $\bar{S}$ represent the averages of the experimental values $E_{i}$ and simulation values $S_{i}$, respectively, for the displacement at the same force, and *N* is the total number of data points (*N* = 75).

Hence, the force–displacement loop resulting from the partial unloading and reloading during indentation was used as the target for the optimization. For determining the material parameters that meet the given objective (i.e., that minimize the value of NMSE), a genetic algorithm [33] was used because it does not require a good initial guess for the target parameters. This algorithm generates the first iteration, which contains certain material parameter sets (one set contains four parameters for kinematic hardening and two for isotropic hardening) for the identification process. The force–displacement loop from the simulation was taken out using a postprocessing script written in Python 3. Based on the fitness results obtained at the end of the first iteration, the algorithm updates the material parameters in the subsequent iterations as the optimization loop continues. In each iteration, the algorithm calculates the fitness of the obtained force–displacement loop with the experimental force–displacement loop by using NMSE. This optimization loop continues until the convergence criterion (i.e., NMSE = 3 × 10^−5^) is met or the maximum allowed iterations are reached. The yield stress and Young’s modulus are kept constant at 1060 MPa and 204 GPa, respectively, based on monotonic stress–strain experimental data. It is known that the yield strength cannot be uniquely determined only based on force–displacement curves with sharp indenters. Hence, this material parameter must be assumed as known and can be determined by other methods, e.g., tensile tests or other inverse methods based on indentation (e.g., see [1,2,3,4,5,6,7,8,9,10,11]).

Two kinds of optimization procedures were studied in this work: “objective function 1” includes a free optimization of the objective function defined in Equation (4) until the convergence criterion is reached; “objective function 2” is also based on the objective function of Equation (4), but the minimization occurs under the side condition that the height of the force–displacement loop is restricted to the experimentally found value, i.e., ΔF_sim_ = ΔF_exp_.

## 4. Results and Discussion

### 4.1. Method Development

The experimental force–displacement loop of the first indentation cycle is used as a target curve along with ΔF. The material parameters (see Table 1) obtained after the optimization with this strategy show a good agreement for the complete indentation cycle: The normalized mean square error (NMSE) between the simulated force–displacement loop and the experimental force–displacement loop is 2.0 × 10^−5^.

**Figure 6:**
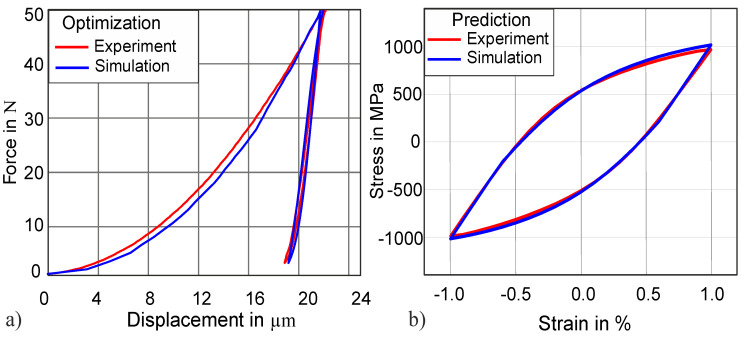
(**a**) Complete cycle of the force–displacement curve from indentation, with normalized mean square error (NMSE) = 2.0 × 10^−5^. (**b**) Predicted uniaxial stress–strain hysteresis with a plastic work error of 2.5%.

To achieve a comparison with experimentally determined hysteresis from fatigue tests, the hysteresis under a tensile–compressive load is predicted in the next step by using the parameters from Table 1. Comparing this prediction to the experimental results reveals, with a plastic work error of 2.5%, a quite good accuracy, as can be seen in Figure 6. In the scope of this study, the identified material parameters of cyclic macroindentation are used to predict the complete uniaxial stress–strain hysteresis for the first time.

**Figure 7:**
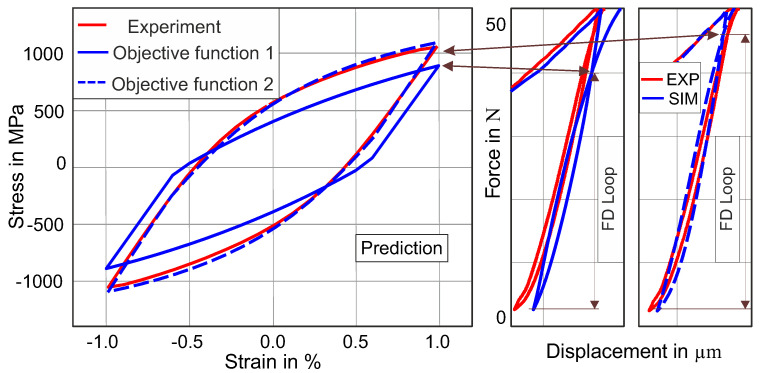
Effect of ΔF on the uniaxial stress–strain hysteresis prediction. The solid blue force-displacement (FD) loop displays the fitting of the FD loop to the blue experimental FD loop by using objective function 1, while the dotted solid stress–strain hysteresis is the prediction of stress–strain hysteresis. Similarly, the dotted blue FD loop shows the fitting of the FD loop by using objective function 2, while the dotted blue stress–strain hysteresis represents the prediction of the stress–strain hysteresis.

There is a clear relationship between the ΔF value measured by cyclic indentation and the uniaxial stress–strain hysteresis. This relationship is qualitatively investigated in this study. In Figure 7, the results from the two objective functions can be seen.

By using the “objective function 1”, the comparison of the simulated and experimental force–displacement loops seems to be in an acceptable range of agreement (see Figure 7, solid blue FD loop), with NMSE 3.0 × 10^−5^; nevertheless, the value of ΔF from the simulation is lower than the experimental ΔF, which has a direct impact on the uniaxial stress–strain hysteresis prediction. The inclusion of the ΔF into “objective function 2” leads to a better prediction of the uniaxial stress–strain hysteresis, which is shown by the dotted blue line hysteresis in Figure 7.

It can be observed that if the simulated ΔF has a lower value than the experimental ΔF, the prediction of the uniaxial stress–strain hysteresis reveals a larger disagreement (plastic work error = 20%) between the experimental and the simulated stress–strain hysteresis. On the other hand, when the value of ΔF is comparable to the experimental ΔF value, the uniaxial stress–strain hysteresis provides an acceptable prediction (plastic work error = 2.0%), as demonstrated by the dotted solid curve in Figure 7.

**Figure 8:**
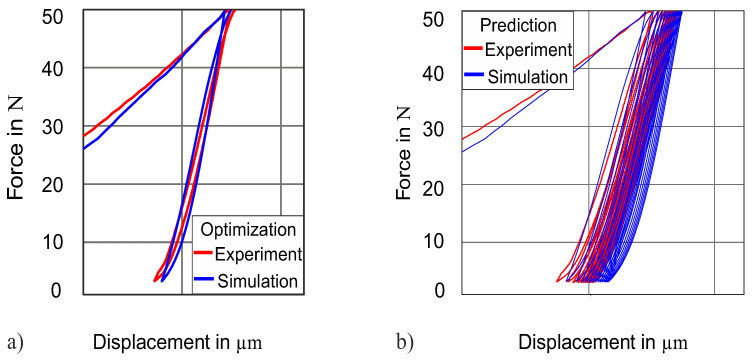
(**a**) Simulated force–displacement loop. (**b**) Predicted force–displacement for 13 cycles.

From now on, we will only present the results obtained by “objective function 2” after optimization. As the Chaboche material model is also capable to capture the ratcheting behavior in cyclic loading, further simulations are performed with multiple cycles of indentation to compare the experimental ratcheting effect of force–displacement by using the identified parameters from the complete indentation cycle. The ratcheting observed in simulation and experiment is slightly overestimated. In Figure 8b, force–displacement curves for 13 consecutive cycles are compared with the experiment.

### 4.2. Validation

**Figure 9:**
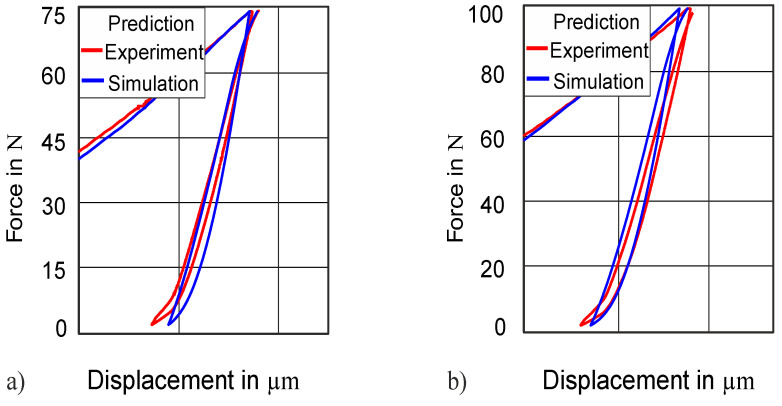
Validation of the method at higher force amplitudes: (**a**) predicted force–displacement at 75 N; (**b**) predicted force–displacement at 100 N.

The material parameters, which have been identified at a 50 N force amplitude, are also tested at higher forces of 75 and 100 N to check the validity of the obtained parameters. The comparison of the simulation curve and the experimental curve at higher force amplitudes reveals that they are also in good agreement at 75 N force amplitude (NMSE = 3.3 × 10^−4^; Figure 9a) and 100 N force amplitude (NMSE = 1.6 × 10^−4^; Figure 9b).

**Figure 10:**
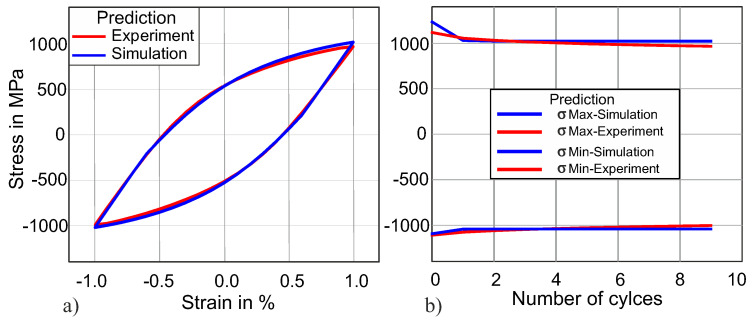
(**a**) Predicted uniaxial stress–strain hysteresis for the 10th cycle. (**b**) Stress amplitude over the number of cycles for the first 10 cycles.

Figure 10a demonstrates the comparison of experimental and simulated uniaxial stress–strain hystereses for the 10th cycle of the same material. The red hysteresis, which is obtained by using the above-obtained fitted material parameters from the cyclic indentation force–displacement curve, shows a quite good agreement (plastic work error = 3.5%) with the experimental hysteresis. Figure 10b demonstrates the maximum and minimum stress on the vertical axis, while the horizontal axis displays the cycle number.

It is evident from Figure 10 that the material parameters obtained from the cyclic indentation force–displacement curve can predict higher uniaxial stress–strain hysteresis very accurately, and the difference is less than 4%. This uniaxial stress–strain hysteresis is obtained without any initial input from uniaxial stress–strain hysteresis. The uniaxial stress–strain hysteresis has a key role in determining material fatigue life. As already mentioned, performing fatigue experiments is quite expensive both in terms of cost and time, and performing indentation tests is quite easy and requires fewer resources. By using this technique of identifying kinematic hardening material parameters, the need to perform fatigue experiments will be required only for the validation process.

### 4.3. Transferability of the Method

#### 4.3.1. Transferability to Higher Force Amplitude (75 N)

**Figure 11:**
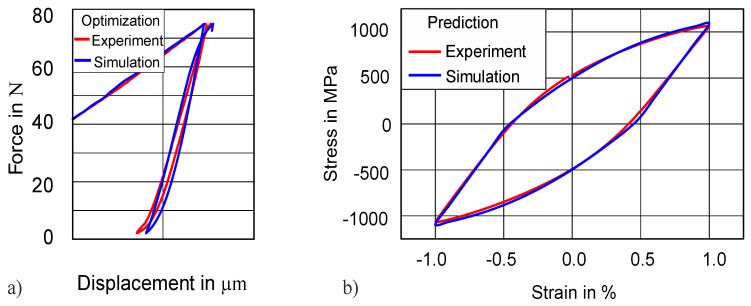
Transferability of method: (**a**) indentation of 38 HRC at 75 N; (**b**) prediction of stress–strain hysteresis of 38 HRC.

Until now, we have used a 50 N indentation force amplitude for identifying material parameters and then predicted the uniaxial stress–strain hysteresis by using these identified parameters. To check the robustness and transferability of our method, we have decided to also fit the 75 N indentation curve and to try to predict the uniaxial stress–strain hysteresis by using these parameters. The rest of the optimization setting and procedure were kept the same as explained before. Figure 11a displays the comparison of the cyclic force–displacement curve, while Figure 11b demonstrates the comparison of the uniaxial stress–strain between simulation and experiment. The results are in good agreement (NMSE = 4.0 × 10^−5^) between the experimental and the simulated curves for both force–displacement and uniaxial stress–strain hysteresis. The parameters obtained after the simulation are reported in Table 2 and not much different from the parameters obtained for the 50 N force amplitude.

#### 4.3.2. Transferability to Higher Hardness (47 HRC)

**Figure 12:**
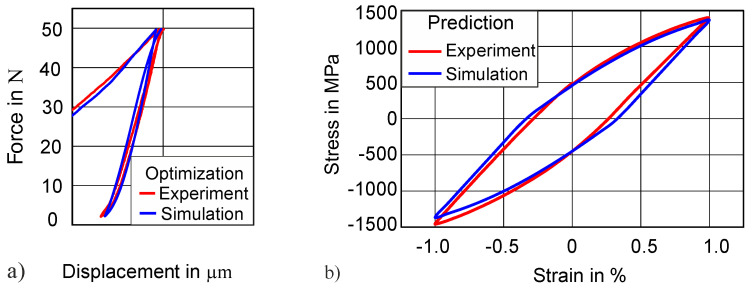
Transferability of method: (**a**) simulated indentation force–displacement at 50 N for 47 HRC; (**b**) prediction of uniaxial stress–strain hysteresis of 47 HRC.

Similarly, the force–displacement loop at 50 N force amplitude of 47 HRC hardness is used for material parameter identification by the inverse method. The value of yield strength used (1400 MPa) was obtained from the monotonic loading experiments. The results of the force–displacement curve after the optimization show a good agreement between the experimental and simulated force–displacement loops, as shown in Figure 12. The prediction of the uniaxial stress–strain curve (by using the material parameters from Table 3) also depicts a sufficient agreement between simulation and experiment. The difference in energy dissipation between the experiment and the prediction is 4.5%.

#### 4.3.3. Transferability for Other Material (Cu)

**Figure 13:**
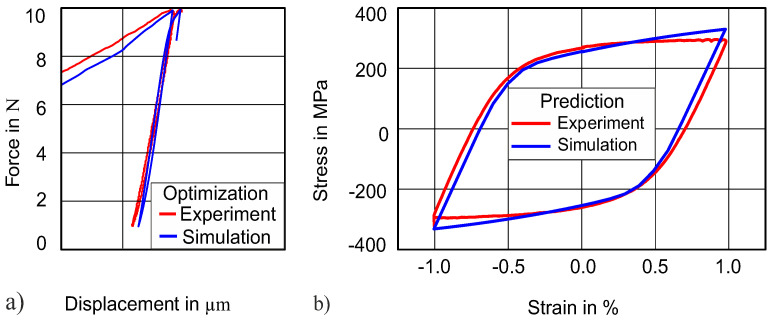
Transferability of method: (**a**) simulated indentation force–displacement of Cu at 10 N; (**b**) prediction of uniaxial stress–strain hysteresis.

The extensive study of using different force amplitudes and different hardnesses has been done with our method for martensitic steel in the previous section. In this section, the aim is to test our methodology on a different metallic material. For this purpose, copper (Cu), a relatively softer material is selected, and therefore, instead of using a 50 N force amplitude, a smaller force amplitude of 10 N is applied for cyclic indentation. The optimization is performed by using the same setup with two backstress terms (Table 4), and the results after optimization are shown in Figure 13. Figure 13a demonstrates the comparison of the force–displacement at 10 N between simulation and experiment, while Figure 13b shows the predicted uniaxial stress–strain at 1% total strain amplitude. It can be seen from Figure 13a that the force–displacement has quite a good fit after optimization. The same is true when we predict the uniaxial stress–strain hysteresis by using these identified parameters and compare it with the experimental uniaxial stress–strain hysteresis. The difference in dissipated energy and between predicted and experimental stress–strain hysteresis is only 3.5%.

## 5. Conclusions

A novel hybrid method for the inverse analysis of fatigue properties of metals has been introduced. The method combines cyclic Vickers indentation experiments and finite element simulations in an inverse method, by which the material parameters are determined in an iterative way by an optimization scheme. It has been demonstrated that this method can be used to determine the parameters of the Chaboche model for kinematic hardening. Based on these parameters, the model has been successfully employed to predict the cyclic stress–strain responses of a tempered martensitic steel, SAE 4150 (German denomination 50CrMo4), with different heat treatments and of technically pure copper. The error in the parameters determined with the inverse method has been evaluated as less than 4% on average. It has been observed that the difference between the maximum and minimum force of the force–displacement loop obtained from cyclic indentation has a direct correlation with the stress amplitude of the hysteresis loop measured in strain-controlled uniaxial fatigue tests; thus, it plays a crucial role in predicting the uniaxial stress–strain hysteresis accurately. By applying the method to high-strength martensitic steel, on which it has been validated for different maximum forces, and also to technically pure copper, its validity has been demonstrated for a wide variety of materials and process parameters. The prediction of a complete cyclic stress–strain curve by using data from cyclic indentation has great potential to reduce time- and cost-intensive fatigue experiments and can thus open a new and economic way to predict the fatigue life of materials with a quasi-nondestructive test method.

## Figures and Tables

**Table 1:** Identified material parameters for 50CrMo4 (38 HRC) after fitting of force–displacement at 50 N.

Symbol	Value
C_1_ (MPa)	262,197
γ_1_	373
C_2_ (MPa)	4714
γ_2_	0.25
Q (MPa)	−575
b	262

**Table 2:** Identified material parameters for 50CrMo4 (38 HRC) after fitting of force–displacement at 75 N force amplitude.

Symbol	Value
C_1_ (MPa)	257,503
γ_1_	354
C_2_ (MPa)	3663
γ_2_	0.2837
Q (MPa)	−611
b	163

**Table 3:** Identified material parameters for 50CrMo4 (47 HRC) of force–displacement at 50 N force amplitude.

Symbol	Value
C_1_ (MPa)	337,885
γ_1_	374
C_2_ (MPa)	6681
γ_2_	2.3
Q (MPa)	−724
b	273

**Table 4:** Identified material parameters of the force–displacement loop for Cu at 10 N force amplitude.

Symbol.	Value
C_1_ (MPa)	154,790
γ_1_	2,257
C_2_ (MPa)	11,586
γ_2_	82
Q (MPa)	−12
b	47

## References

[1] Huber N., Tsakmakis C. (1999). Determination of constitutive properties from spherical indentation data using neural networks. Part I: The case of pure kinematic hardening in plasticity laws. J. Mech. Phys. Solids.

[2] Huber N., Tsakmakis C. (1999). Determination of constitutive properties from spherical indentation data using neural networks. Part II: Plasticity with nonlinear isotropic and kinematic hardening. J. Mech. Phys. Solids.

[3] Wymysłowski A., Dowhań Ł. (2013). Application of nanoindentation technique for investigation of elasto-plastic properties of the selected thin film materials. Microelectron. Reliab..

[4] Oliver W.C., Pharr G.M. (2004). Measurement of hardness and elastic modulus by instrumented indentation: Advances in understanding and refinements to methodology. J. Mater. Res..

[5] Peirce D., Asaro R.J., Needleman A. (1983). Material rate dependence and localized deformation in crystalline solids. Acta Metall..

[6] Hyung-Yil L. (2002). Ball Indenter Utilizing Fea Solutions for Property Evaluation. https://patents.google.com/patent/WO2003010515A1.

[7] Suresch A., Alcala S., Giannakopoulos J. (1996). Depth Sensing Indentation and Methodology for Mechanical Property Measurements. https://patents.google.com/patent/WO1997039333A2.

[8] Suresh T.A., Dao S., Chollacoop M., Van N., Venkatesh K.V. (2002). Systems and Methods for Estimation and Analysis of Mechanical Property Data. https://patents.google.com/patent/WO2002073162A2.

[9] Fontanari V., Beghini M., Bertini L. (2004). Method and Apparatus for Determining Mechanical Features of a Material with Comparison to Reference Database. https://patents.google.com/patent/WO2006013450A2/en.

[10] Schmaling B., Hartmaier A. (2011). Method for Testing Material, particularly for Hardness Testing, Involves Producing Impression in to Be Tested Material in Experimental Manner with Test Body with Known Geometry and with Known Test Load. https://patents.google.com/patent/DE102011115519A1/de.

[11] Broitman E. (2017). Indentation Hardness Measurements at Macro-, Micro-, and Nanoscale: A Critical Overview. Tribol. Lett..

[12] Strzelecki P. (2014). Analytical Method for Determining Fatigue Properties of Materials and Construction Elements in High Cycle Life.

[13] Murakami Y. (1989). Effects of small defects and nonmetallic inclusions on the fatigue strength of metals. JMSE Int. J..

[14] Bandara C.S., Siriwardane S.C., Dissanayake U.I., Dissanayake R. (2015). Developing a full range S-N curve and estimating cumulative fatigue damage of steel elements. Comput. Mater. Sci..

[15] Bandara C.S., Siriwardane S.C., Dissanayake U.I., Dissanayake R. (2016). Full range S-N curves for fatigue life evaluation of steels using hardness measurements. Int. J. Fatigue.

[16] Strzelecki P., Tomaszewski T. (2017). Analytical models of the S-N curve based on the hardness of the material. Procedia Struct. Integr..

[17] Lyamkin V., Starke P., Boller C. Cyclic indentation as an alternative to classic fatigue evaluation. Proceedings of the 7th International Symposium on Aircraft Materialsno.

[18] Faisal N.H., Prathuru A.K., Goel S., Ahmed R., Droubi M.G., Beake B.D., Fu Y.Q. (2017). Cyclic Nanoindentation and Nano-Impact Fatigue Mechanisms of Functionally Graded TiN/TiNi Film. Shape Mem. Superelasticity.

[19] Haghshenas M., Klassen R.J., Liu S.F. (2017). Depth-sensing cyclic nanoindentation of tantalum. Int. J. Refract. Met. Hard Mater..

[20] Prakash R.V. (2010). Evaluation of fatigue damage in materials using indentation testing and infrared thermography. Trans. Indian Inst. Met..

[21] Prakash R.V. (2017). Study of Fatigue Properties of Materials through Cyclic Automated Ball Indentation and Cyclic Small Punch Test Methods. Key Eng. Mater..

[22] Xu B.X., Yue Z.F., Chen X. (2009). Numerical investigation of indentation fatigue on polycrystalline copper. J. Mater. Res..

[23] Schäfer B., Song X., Sonnweber-Ribic P., Hassan H.U., Hartmaier A. (2019). Micromechanical Modelling of the Cyclic Deformation Behavior of Martensitic SAE 4150—A Comparison of Different Kinematic Hardening Models. Metals (Basel).

[24] Kramer H.S., Starke P., Klein M., Eifler D. (2014). Cyclic hardness test PHYBALCHT - Short-time procedure to evaluate fatigue properties of metallic materials. Int. J. Fatigue.

[25] DIN EN ISO 6507-2 (2005). Metallic Materials—Vickers Hardness Test—Part 2: Verification and Calibration of Testing Machines.

[26] Mises R.V. (1913). Mechanik der festen Körper im plastisch- deformablen Zustand. Nachrichten von der Gesellschaft der Wissenschaften zu Göttingen, Mathematisch-Physikalische Klasse.

[27] Srnec Nova J., Benasciutti D., De Bona F., Stanojević A., De Luca A., Raffaglio Y. (2016). Estimation of Material Parameters in Nonlinear Hardening Plasticity Models and Strain Life Curves for CuAg Alloy. IOP Conf. Ser. Mater. Sci. Eng..

[28] Chaboche J.L.L. (1989). Constitutive equations for cyclic plasticity and cyclic viscoplasticity. Int. J. Plast..

[29] Lemaitre J., Chaboche J.-L. (1990). Mechanics of Solid Materials.

[30] Frederick C.O., Armstrong P.J. (2007). A mathematical representation of the multiaxial Bauschinger effect. Mater. High Temp..

[31] Sajjad H.M., Hanke S., Güler S., ul Hassan H., Fischer A., Hartmaier A. (2019). Modelling cyclic behaviour of martensitic steel with J2 plasticity and crystal plasticity. Materials.

[32] About LS-OPT—DYNAmore GmbH. https://www.dynamore.de/de/produkte/opt/ls-opt.

[33] Chaparro B.M., Thuillier S., Menezes L.F., Manach P.Y., Fernandes J.V. (2008). Material parameters identification: Gradient-based, genetic and hybrid optimization algorithms. Comput. Mater. Sci..

